# Supplementary material for: Analysis of HPV Integrations in Mexican Pre-Tumoral Cervical Lesions Reveal Centromere-Enriched Breakpoints and Abundant Unspecific HPV Regions
Source: Int J Mol Sci. 2021 Mar 22;22(6):3242. doi: 10.3390/ijms22063242 (PMC8005155; doi:10.3390/ijms22063242)
Supplement: Supplementary file 1 [file ijms-22-03242-s001.zip › Supplementary Figure 1.pdf]

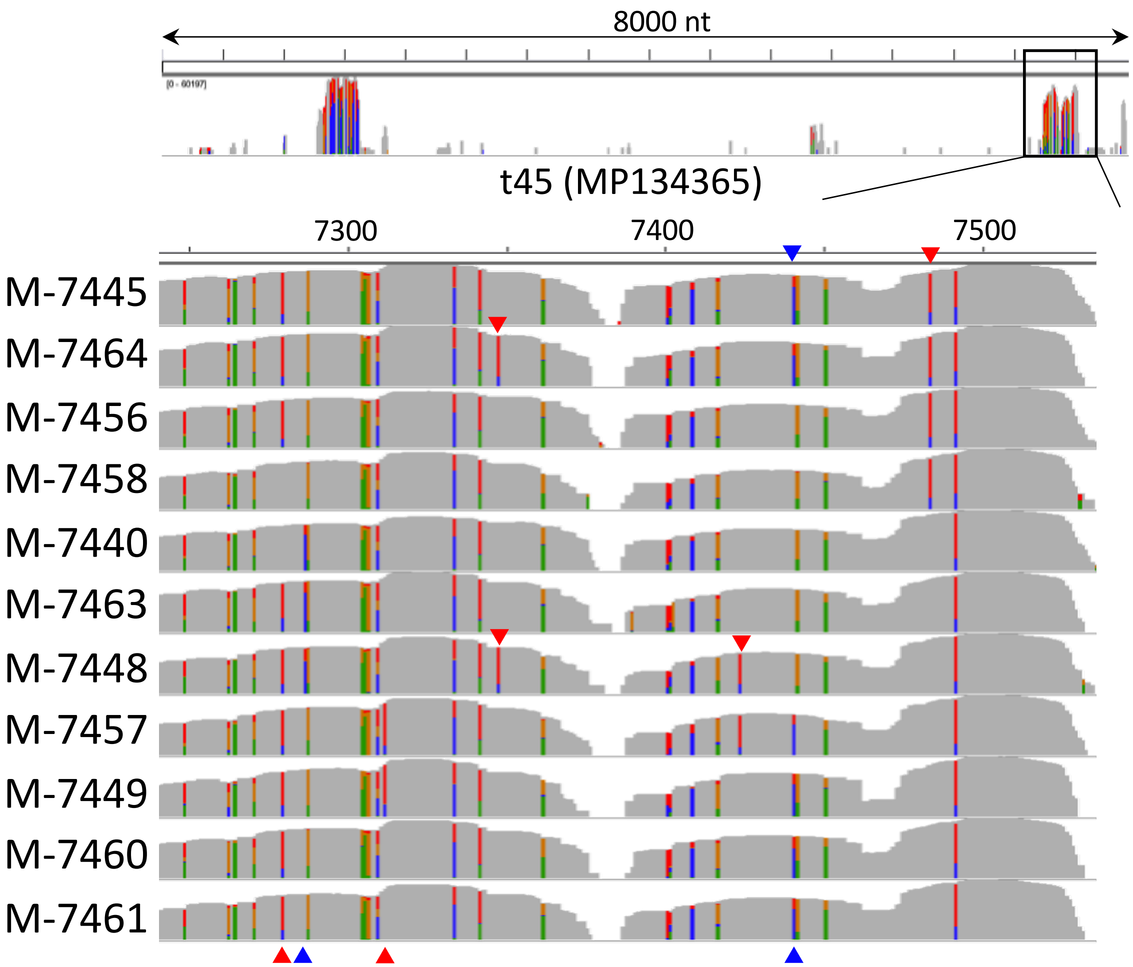

Supplementary Figure 1. Samples and reads mapped to t45. Top panel shows the reads for a representative sample. Bottom panel show the read depth plots and variants using IGV for a region around 7400 nt. The vertical axis is in logarithm scale. The pattern of variants is specific for most samples. Samples M-7460 and M-7461 looks the same in this region but the second region around 1,500nt show clear differences.
