# Supplementary material for: Analysis of HPV Integrations in Mexican Pre-Tumoral Cervical Lesions Reveal Centromere-Enriched Breakpoints and Abundant Unspecific HPV Regions
Source: Int J Mol Sci. 2021 Mar 22;22(6):3242. doi: 10.3390/ijms22063242 (PMC8005155; doi:10.3390/ijms22063242)
Supplement: Supplementary file 1 [file ijms-22-03242-s001.zip › Supplementary Table 1.docx]

**Supplementary Table 1**: HPV genotypes identified by qPCR and NGS. Samples and genotypes in which the two techniques coincide are marked in bold.

| Sample | Pap smear | HPV type by qPCR | HPV Types by NGS |
| --- | --- | --- | --- |
| M-7440 | CC | 16, 58 | t45 |
| M-7443 | LSIL | **18** | **18**, 74 |
| M-7445 | LSIL | **68** | t45, **68**, NA440, NA448, |
| M-7447 | LSIL | 39, **52,** 56, 68 | 44, t45, **52,** **t52**, t66, 74, 87, |
| M-7448 | ASCUS | 68, 56, 52 | 6, t45 |
| M-7449 | Normal | 18, 39, 51, 52 | t45 |
| M-7450 | ASCUS | 39, 35, 51, **52, 56**, 68, 56 | 11, **52, t52, t56, 56,** NA450 |
| M-7452 | ASCUS | 16, 18, **59** | t45, 53, **59**, 89 |
| M-7454 | LSIL | 52 | 11, 30, 42, t45, 51 |
| M-7455 | Unknown | **45**, 58 | 16, 35**, t45**, NA446 |
| M-7456 | HSIL | **16**, 31 | **16**, t45, |
| M-7457 | LSIL | 39, 56 | 6, t45, 51, 90 |
| M-7458 | LSIL | 16, 18, 68 | 6, t45, 86 |
| M-7459 | HSIL | **31** | **31**, t45 |
| M-7460 | LSIL | 39**, 51,** 59 | 34, t45**, 51**, 70 |
| M-7461 | LSIL | 39, 51, 59 | 44, t45, 53, 54 |
| M-7462 | LSIL | **51** | **51,** 54 |
| M-7463 | LSIL | **16** | **16**, 40, t45, NA446 |
| M-7464 | LSIL | **31**, 39, 52, 56, 59 | 11, **31,** 34, t45 |
| M-7465 | Unknown | **66** | t45, **66, t66,** NA451 |
| M-7467 | Normal | 66 | 11, t45 |
| M-7470 | LSIL | 18, **33,** 39, **52** | **33, t33,** t45, **52, t52,** 53, NA436 |
| M-7471 | Normal | **39**, 51, **52**, 66 | **39, t39,** 42, t45, **52, t52,** 66, 73, 82, NA447, NA449 |
| M-7472 | LSIL | 56 | t45, 51 |
